# Emerging Multifunctional NIR Photothermal Therapy Systems Based on Polypyrrole Nanoparticles

**DOI:** 10.3390/polym8100373

**Published:** 2016-10-20

**Authors:** Mozhen Wang

**Affiliations:** CAS Key Laboratory of Soft Matter Chemistry, Department of Polymer Science and Engineering, University of Science and Technology of China, Hefei 230026, China; pstwmz@ustc.edu.cn; Tel.: +86-551-6360-0843

**Keywords:** polypyrrole, near infrared light, photothermal therapy, drug release, imaging, nanoparticle, cancer therapy

## Abstract

Near-infrared (NIR)-light-triggered therapy platforms are now considered as a new and exciting possibility for clinical nanomedicine applications. As a promising photothermal agent, polypyrrole (PPy) nanoparticles have been extensively studied for the hyperthermia in cancer therapy due to their strong NIR light photothermal effect and excellent biocompatibility. However, the photothermal application of PPy based nanomaterials is still in its preliminary stage. Developing PPy based multifunctional nanomaterials for cancer treatment in vivo should be the future trend and object for cancer therapy. In this review, the synthesis of PPy nanoparticles and their NIR photothermal conversion performance were first discussed, followed by a summary of the recent progress in the design and implementation on the mulitifunctionalization of PPy or PPy based therapeutic platforms, as well as the introduction of their exciting biomedical applications based on the synergy between the photothermal conversion effect and other stimulative responsibilities.

## 1. Introduction

Over the past decades, various environmental-responsive smart polymeric materials have been constantly developed to meet the diverse needs in the biomedical field, especially in the tailor-made design of smart delivery and release systems for the cancer therapy [1,2,3,4,5,6,7,8,9,10,11,12,13,14,15]. On the one hand, compared with the conventional small organic molecular carriers or inorganic nanoparticles, polymers can maintain the stability and integrity of the incorporated substances for longer period of time [16,17] and exhibit good biocompatibility and minimal unwanted side effects on other organs and/or tissues [18]. However, more importantly, the myriad chemical structures of polymer chains can provide not only chemical or physical connection and a protective “coat” to the desired therapeutic substances, but also a variety of responsive units for various internal stimuli (such as pH [19,20], temperature [21,22], enzyme [23], and redox environment [24,25,26]) and external stimuli (such as ultrasound [27,28], radiofrequency [29], light [30,31,32,33,34], and magnetic field [35,36]). Practically, the biosafety and the efficiency of internal stimuli closely relate to the detailed in vivo environments, which may have large individual variations. However, there is no dependence on individuals if using external stimuli, such as light. Light can be operated remotely, easily regulated and focused, which makes it much easier for the light-triggered therapy platforms to achieve precise control of treatment timings and locations, and cause relatively less damage to healthy tissues [37,38,39,40,41]. Therefore, light-sensitive polymer or polymer composite platforms for biomedical application have been extensively explored recently.

However, biological tissues have the ability to absorb and scatter the light in a certain energy range, e.g., they have the strong absorption for UV-visible light (λ = 350–650 nm) and red light (λ > 1000 nm). This results in a rather limited tissue penetration depth of UV or visible light, which becomes a major obstacle for their in vivo applications [42,43]. Besides, prolonged exposure to UV light also can cause severe cellular photodamage [44]. On the contrary, biological tissues have a minimum absorption and scattering for near infrared (NIR) light (λ = 650–1000 nm), which makes the tissue penetration depth of NIR light be about an order of magnitude greater than that of UV-visible light [45] (so-called transparency “therapeutic window” for biological applications). At the same time, NIR light exhibits no adverse effect on the physiological function of living tissues (under a moderate light intensity) and no background fluorescence [46,47]. In particular, NIR light has been discovered to have the ability to generate heat, i.e., photothermal effect, when it interacts with many inorganic nanoparticles [48,49,50,51,52,53,54] and conducting polymers such as polypyrrole (PPy), polyaniline (PANI), and polydopamine (PDA) [55,56,57,58], which means the simultaneous delivery of heat and chemotherapy can be naturally combined through the rational design of NIR-light sensitive conducting polymer systems. Therefore, some researchers even predict the NIR-light-triggered therapy platforms to be a new and exciting possibility for clinical nanomedicine applications [59].

PPy was first documented in the early twentieth century with a name of “pyrrole black” obtained from its origin, an insoluble black precipitate in an acidic pyrrole/H_2_O_2_ aqueous solution. Its semiconducting properties had been highlighted until the pioneering work of McNeil et al. in 1963 [60] and Dall’Olio et al. in 1968 [61] on the electrochemical synthesis method of PPy, which triggered the following numerous explorations on developing its conductive property and the related applications. Ultimately, PPy becomes a famous member in the conducting polymer family [62]. In addition to its remarkable electrical properties, PPy has been proven to be biocompatible and may be introduced into a human body without any harmful effect on health [63,64], making it now an attractive electro-responsive material in a center of attention for biomedical engineering [65,66,67]. However, with the rapid development of the biomedical science and the polymer synthetic technique as well as more detailed understanding of physico-chemical properties of PPy, the rational design and synthesis on the PPy-based integrated therapeutic platforms that possess synergistic effect of chemotherapy, gene therapy, and/or photothermal therapy (PTT) have received great attention since they can achieve effective and powerful strategies for “precise” cancer therapy with as less the drug dosages and side effects as possible [68,69,70]. Meanwhile, the photothermal effect commonly can be visually characterized by photothermal imaging (PTI) and/or photoacoustic imaging (PAI), which means that PPy-based therapeutic platforms have the potential to achieve the visualization of therapy [71].

Thus in this review, we will summarize recent progresses in the design and implementation on the multifunctionalization of PPy or PPy-based therapeutic platforms, introduce their exciting biomedical applications based on the synergy between the photothermal conversion effect and other stimulative responsibility, and finally discuss the challenges and prospects of this rapidly growing field.

## 2. Synthesis and NIR-Triggered Photothermal Conversion Performance of PPy Nanoparticles (PPy NPs)

**Figure 1:**
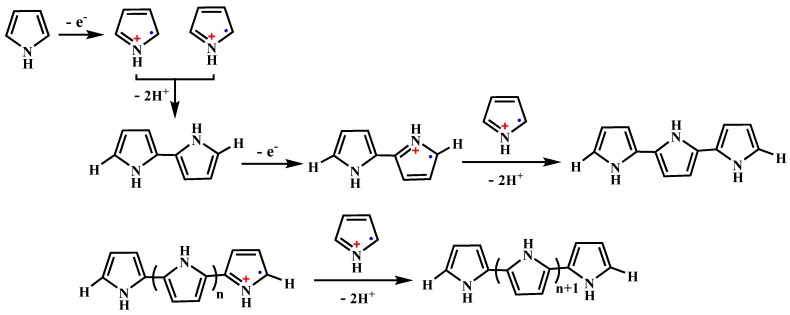
Oxidative polymerization of pyrrole to polypyrrole.

Based on the current understanding, PPy is obtained by the cationic radical (i.e., oxidative) polymerization of pyrrole via a pseudo-polycondensation mechanism as shown in Figure 1 [72,73]. It starts from a one electron oxidation of pyrrole to a radical cation. Two pyrrole radical cations couple to form the 2,2′-bipyrrole, which subsequently couples with another radical cation. This process is then repeated until longer chains are formed. Practically, the oxidation can be achieved electrochemically at an electrode through the application of an oxidizing potential [74,75] or chemically in solution, using a chemical oxidant (e.g., (NH_4_)_2_S_2_O_8_, FeCl_3_, H_2_O_2_) [76,77,78]. The morphology of PPy greatly depends on the synthesis method and conditions. The electrochemical oxidative polymerization of pyrrole favors to obtain PPy bulk materials, such as conductive film or else. However, in order to be used as in vivo delivery and therapy systems, the size of the particulate systems should be carefully controlled in a range of about 30 and 300 nm because before reaching the target site, the particles undergo a biodistribution step possibly after crossing epithelial barriers and travelling through the vascular bed [79]. The preparation of PPy by chemical oxidation polymerization had been widely investigated in various organic solvent and aqueous media using water-soluble oxidative agent since 1960’s. However, in the early stage, the morphological control of the obtained PPy products received little concern compared with their conductive properties. In recent twenty years, with the discovery of the NIR photothermal effect and the widely application in biomedical field, researchers have been devoted to explore more precise synthesis routes and conditions for morphology- and size-controllable PPy NPs based on the chemical oxidation polymerization mechanism.

**Figure 2:**
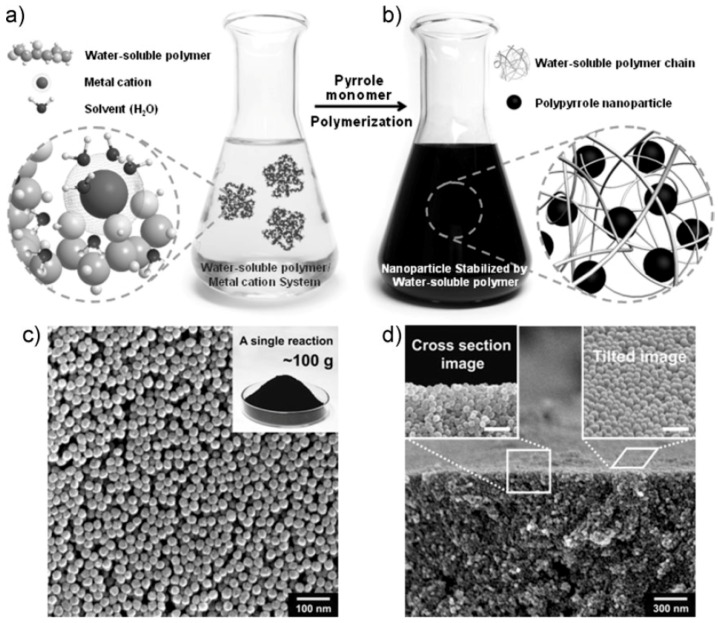
(**a**,**b**) Schematic illustration of the formation process and mechanism of PPy NPs in an aqueous dispersion of water-soluble polymer; (**c**) SEM images of the resulting PPy NPs (inset: photograph showing a Petri dish containing 100 g of PPy NPs obtained in a single polymerization reaction); (**d**) Tilted and cross-section SEM images of the PPy NPs stacked on a substrate (scale bar in insets: 100 nm). Reproduced with permission from [82].

Armes et al. [78,80] had developed a series of work on the preparation of PPy from an aqueous media and pointed out that monodispersed spherical PPy NPs can be prepared based on the chemical oxidative polymerization of pyrrole in the aqueous solution using FeCl_3_ as the oxidant and water soluble polymer (polyvinylpyrrolidone (PVP) and polyvinyl alcohol (PVA)) as the stabilizer. Woo et al. [81] and Hong et al. [82] investigated the synthesis conditions more detailed, individually using PVP and PVA as the stabilizer respectively. The overall synthetic procedure could be schematically illustrated in Figure 2a,b. Here, the water-soluble polymer was considered to function in two ways: one is complexing with iron cations through the interactions between polar groups and cations, the other is coating around on the newly formed PPy NPs to prevent the particle aggregation. Therefore, after the polymerization, the resulting PPy NPs could be dispersed stably in water or other polar organic solvents such as alcohol. The nanoparticles obtained with this route have a uniform diameter of average size ranging from 20 to 100 nm (Figure 2c,d), depending on the molecular weight and the concentration of the water soluble polymers, as well as the concentration of iron cations. In order to obtain the stable PPy NPs, the reaction rate generally needs to be controlled at a relatively low level, thus the temperature should preferably be lower than 20 °C. It is noted that this synthesis route seems to have industry prospect since it can produce PPy NPs in large quantities (subkilogram quantities, Figure 2c inset). It should be noted that the water soluble polymer stabilizer cannot be removed completely from the produced PPy NPs due to the strong affinity between PPy and polymer stabilizer molecule chains. Kobayashi et al. [83] proposed a new method for the synthesis of PPy particles using a water/oil (W/O) emulsion. The W/O emulsion was prepared by stirring the water phase, i.e., the aqueous solution of oxidant ammonium persulfate (APS), and the oil phase, i.e., an organic solvent (such as *n*-hexane, *n*-heptane, *n*-octane, isooctane, *n*-decane, *n*-dodecane, and their mixture) containing the emulsifier, dioctyl sulfosuccinate sodium salt (Aerosol OT). Pyrrole monomer dissolved in the organic solvent was injected in the prepared W/O emulsion. In this polymerization system, the reaction rate was controlled by the diffusion of monomer to a water droplet at room temperature. Thus the morphology and size distribution of the prepared PPy particles were influenced by the characteristics of the emulsion, which is related with the nature of the organic solvent, the volume fraction of the dispersed phase, and the rotation speed. In general, the diameter of PPy particles was about 100 nm. This method seemed simply and could avoid the use of polymer stabilizer on PPy NPs, but for the environment reasons, the post-treatment of the large amount of organic solvent is necessary.

Recently, radiation initiation technique was introduced in the preparation of PPy NPs in replace of chemical initiators. It’s well-known that the radiolysis of pure water can directly produce some active species [84]:

(1) $$H_{2}O\;⇝\;\cdot \mathrm{OH},\;H\cdot ,\;e_{\mathrm{aq}}^{-},\;H_{2}O_{2},\;H_{3}O^{+},\;H_{2}$$

**Figure 3:**
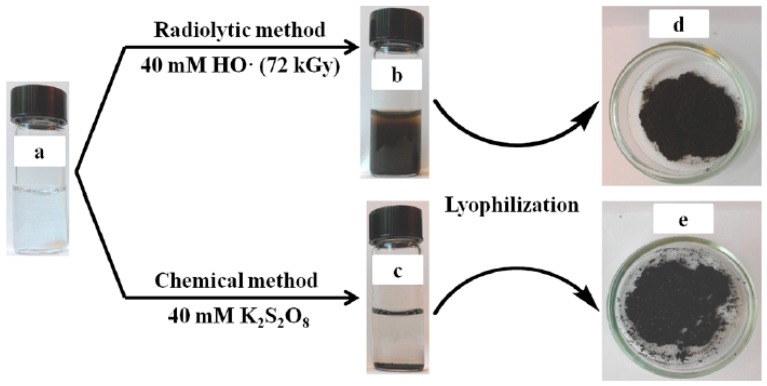
Photographs of pyrrole samples before (**a**); after polymerization (**b**,**c**); and after lyophilization (**d**,**e**). (The right of (**d**) is the SEM image of the sample in (**b**)). Reproduced with permission from [85].

Cui et al. [85] synthesized PPy NPs via γ-ray radiation on the neutral aqueous solution of pyrrole monomer under N_2_O atmosphere, and compared them to the counterpart prepared by chemically initiation, as shown in Figure 3.

The role of N_2_O here was to eliminate the hydrated electrons (e_aq_^−^) produced by water radiolysis through converting them into the strong oxidative species (·OH) according to the following Equation (2) so as to induce the polymerization of pyrrole [86]:

(2) $$H_{2}O+N_{2}O+e_{\mathrm{aq}}^{-}\to N_{2}+{\mathrm{HO}\cdot +\mathrm{HO}}^{-}$$

After 12-hour’s irradiation, the transparent pyrrole solution (the concentration of pyrrole was far lower than its solubility in water (0.9 M)) became a black turbid dispersion, which was relatively stable, and no sedimentation process was observed in this case over few days. The corresponding SEM image of the product showed the presence of low-density globular structures forming polydisperse spherical nanoparticles, the diameter of which was comprised between 80 and 200 nm. But at the case of chemically initiation, black powder deposits at the bottom of the solution (Figure 3c), indicating that they were more hydrophobic than the radiosynthesized ones or could have higher molecular weight since they precipitate. Although no chemical initiator and polymer stabilizer was needed this synthesis system, the morphological controllability of the product was not so good, and N_2_O needs to be very carefully operated for its potential security risk.

Taking the merits and faults of above all synthesis routes into account, most of the current preparation of PPy NPs in biomedical applications are based on the chemically oxidative polymerization in an aqueous medium. The diameter of PPy NPs can be adjusted from 20 to 400 nm by careful control of the experiment conditions.

**Figure 4:**
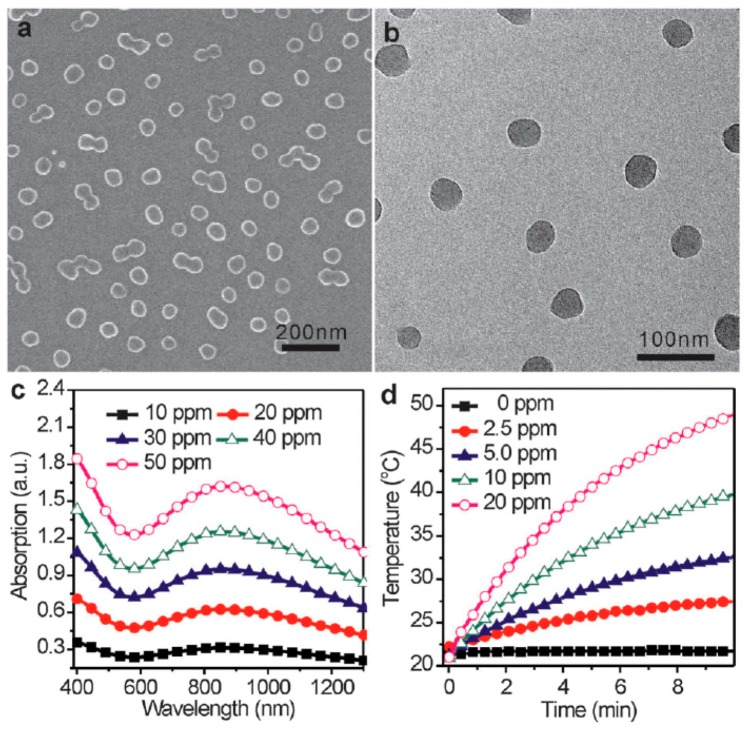
Representative (**a**) SEM and (**b**) TEM images of PPy NPs; (**c**) UV-vis-NIR spectra of PPy NPs at various concentrations; (**d**) Photothermal effect of pure water and PPy NPs with different concentrations upon the irradiation of 1 W·cm^−2^ 808 nm laser. Reproduced with permission from [87].

Zheng et al. [87] synthesized PPy NPs with an average size of ~50 ± 5 nm (Figure 4a,b) via the above mentioned aqueous dispersion polymerization [85] using FeCl_3_ as an oxidation agent and PVP as a capping agent to study NIR phothermal conversion efficiency and the in vivo NIR PTT on 4T1 tumor cells. As is shown in Figure 4c, the synthesized PPy NPs show a strong and broad NIR absorption peak at ~850 nm, which is in accord with the characteristic absorption of bipolaronic metallic state of doped PPy [88]. The extinction coefficient at 808 nm was measured to be 2.38 × 10^10^ M^−1^·cm^−1^. When 1 mL aqueous dispersions of PPy NPs with various concentrations were irradiated with a 808 nm laser with a power density of 1 W·cm^−2^ for 10 min, the temperature of the dispersion increased along with the irradiation time, as recorded in Figure 4d. Then, the photothermal conversion efficiency of PPy NPs could be calculated to as high as 44.7% referring to the method proposed by Roper et al. [89] and Hu et al. [90], while it was reported that hydrophilic Cu_9_S_5_ nanocrystals only with a 25.7% heat conversion efficiency could exhibit an obvious photothermal ablation effect on cancer cells in vivo [90].

**Figure 5:**
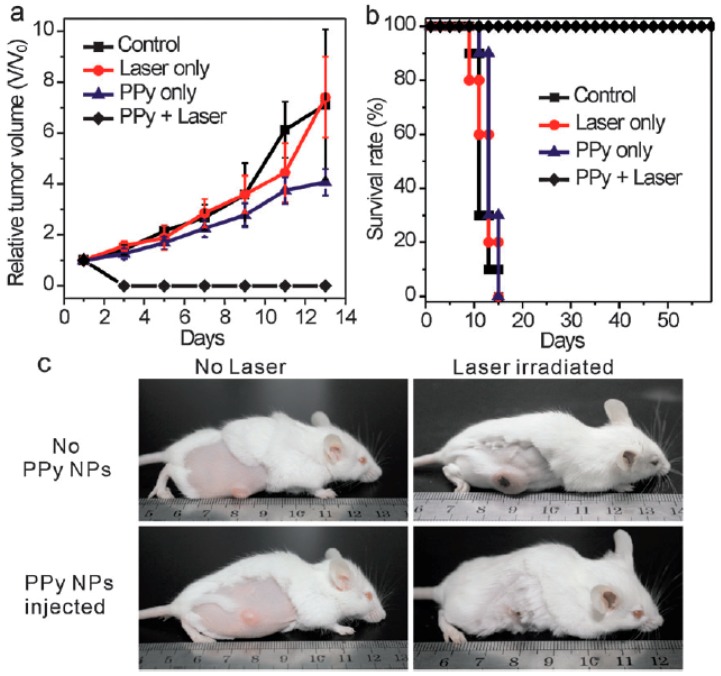
In vivo photothermal therapy study using intravenously injected PPy NPs. (**a**) Tumor growth rates of groups after different treatments; (**b**) Survival curves of mice bearing 4T1 tumor after various treatments; (**c**) Representative photos of tumors on mice after various treatments (only laser treated and PPy + laser treated). Reproduced with permission from [87].

Sequently, the in vitro cytotoxicity of the prepared PPy NPs to human hepatocyte cells QSG-7701 was carried out. It proved that PPy NPs have no appreciable negative effect up to a high concentration of 200 ppm. The result of in vivo photothermal therapy of PPy NPs on the 4T1 tumor model on BALB/c mice is shown in Figure 5, which indicates that only NIR laser irradiation or PPy NPs injection had little influence on the tumor development. But the tumors in the mice treated with the combination of NIR laser irradiation and PPy NPs finally disappeared, and all the mice survived over 60 days, manifesting an excellent in vivo PTT efficacy. In order to take advantage of inductively coupled plasma-atomic emission spectrometry (ICP-AES) to analyze the accumulation of PPy NPs in each organ [91], the authors also prepared core–shell structured SiO_2_@PPy NPs with a similar size to simulate the in vivo behavior of PPy NPs so as to quantitatively assess the biodistribution of PPy NPs. It was found that the highest uptake of the SiO_2_@PPy NPs was in liver (~56% ID·g^−1^), followed by spleen (~10% ID·g^−1^) because these two organs are reticuloendothelial system enriched tissues. The accumulations in heart, kidney, and lung were at a rather low level (<5% ID·g^−1^). The accumulation in tumors was ~5% ID·g^−1^, which may be related with the short blood circulation half-life of the SiO_2_@PPy NPs (only ~12 min).

**Figure 6:**
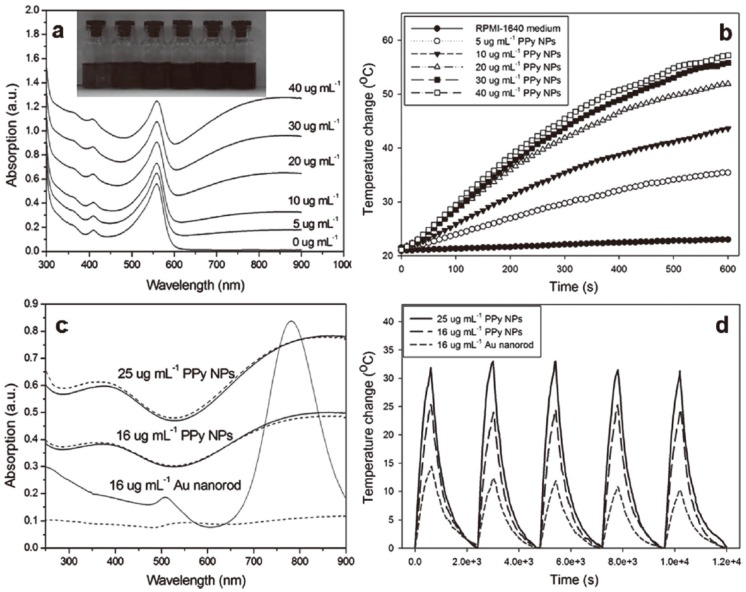
PPy NPs prepared from the oxidative polymerization of Py in an aqueous solution of PVA and FeCl_3_ can act as an efficient and stable photothermal coupling agent. (**a**) UV–vis–NIR spectra of various concentrations of PPy NPs in RPMI-1640 culture medium containing 10% FBS (the inset photograph shows various concentrations of PPy NPs dispersed in RPMI-1640 culture medium, indicating good dispersity); (**b**) Temperature elevation over a period of 10 min of exposure to NIR light (808 nm, 2 W) at various PPy NPs concentrations. RPMI-1640 culture medium was used as a control; (**c**) UV–vis–NIR spectra of PPy NPs and Au nanorods before and after five LASER ON/OFF cycles of NIR light (808 nm, 2 W) illumination (LASER ON time: 10 min; LASER OFF time: 30 min); (**d**) Temperature elevation of PPy NPs and Au nanorods over five LASER ON/OFF cycles of NIR laser irradiation. Reproduced with permission from [92].

Dai’s group [92] also prepared sterically stabilized PPy NPs with a diameter of 46 nm according to a similar synthesis route using PVA as the stabilizer, and evaluated them as a novel class of biocompatible, highly NIR absorbing agent with high photothermal conversion efficiency, as shown in Figure 6. The size stability of the nanoparticles or their aggregates is a key criterion for their use in biomedical applications. As shown in Figure 6a, the prepared PPy NPs were dispersed very well in RPMI-1640 culture medium containing 10% fetal bovine serum (FBS) without any macroscopic aggregates.

**Figure 7:**
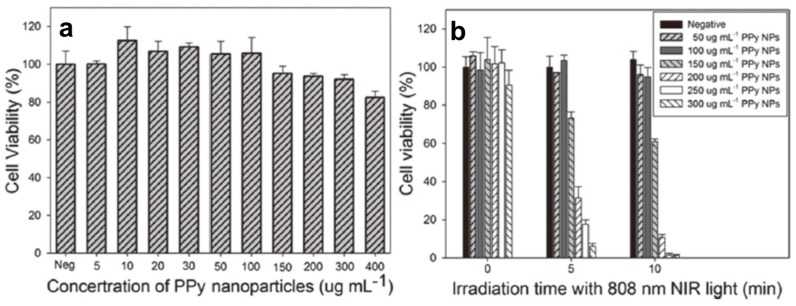
(**a**) Cell viability of HUVECs (human umbilical vein endothelial cells) with 24 h exposure to various concentrations of PPy NPs; (**b**) Cell viability of HeLa cells after treatment with different concentrations of PPy NPs and different NIR laser irradiation time. Reproduced with permission from [92].

The photothermal effect induced by NIR laser irradiation (808 nm, 2 W) in the presence of PPy NPs was investigated by monitoring the temperature change in 3 mL RPMI-1640 culture medium containing various concentrations of PPy NPs, as shown in Figure 6b. At the same time, their photothermal conversion efficiency and NIR photostability were measured and found to be even higher than those of the well-known photothermal agent Au nanorods (Figure 6c,d). MTT assay showed the prepared PPy NPs nearly have no toxic effect on human umbilical vein endothelial cells (HUVECs) (Figure 7a). But significant death of HeLa cells after the combined treatment of PPy NPs and NIR irradiation could be observed in both fluorescence staining assay and MTT assay (Figure 7b), demonstrating the potential application of PPy NPs in the photothermal ablation therapy.

Based on the current research data, PPy NPs has exhibited high photothermal conversion efficiency, photostability, and good biocompatibility, making it potentially become one of the excellent candidate agents for in vivo NIR photothermal cancer therapy. Now, it is universally acknowledged that the non-invasive and targeted photothermal therapy (PTT) using NIR laser has received significant interest as a potentially effective treatment on tumor necrosis, and is considered to be an excellent alternative for cancer therapy in addition to the traditional treatment, such as surgery and chemotherapy. At the same time, the development of multifunctional nanoparticles for the clinical treatment of cancer displays great potential to enhance therapeutic efficacy due to the combination of diagnosis and therapy in a single system [93,94,95]. Thus, more deeper and detailed studies on the integration of other multifunctions with the NIR PTT on the PPy NPs have been underway.

## 3. Multifunctional PPy-Based Therapy Platforms with NIR-Triggered PTT Effect

The combined therapy composed of chemotherapy and photothermal therapy can directly kill the cancer cells by the hyperthermia induced by photothermal agent, and at the same time increase the sensitivity of chemotherapy, resulting in a synergistic enhancement in the therapeutic effects. Practically, there are two forms to combine the chemical drug and photothermal agent in one system. One is loading the drug and photothermal agent on the third-part matrix [96,97,98,99,100,101,102]. The other is enwrapping the drug on the matrix made of the photothermal materials [103,104,105,106,107,108,109,110].

Chiang et al. [98] designed an injectable system of hollow microspheres (HMs) that can rapidly produce localized heat and activate the release of an antibiotic by NIR light. The HMs generated by a capillary fluidic device have a shell of poly(d,l-lactic-*co*-glycolic acid) (PLGA) and an aqueous core comprised of vancomycin (Van) and PPy NPs (with an average diameter of 66.0 ± 10.1 nm, pre-prepared using the chemical oxidation polymerization in an aqueous solution of PVA/Fe^3+^). The detailed preparation operation involves the introduction of three phases at individually adjustable flow rates by syringe pumps. The inner water phase was an aqueous core dispersion containing Van and PPy NPs. The outer water phase was a PVA solution. A PLGA solution in CH_2_Cl_2_ was applied as the middle oil phase. The aqueous PVA solution in an ice bath was the collection phase. The release of Van from PLGA microspheres was typically slowly via diffusion, and it could be accelerated by increasing local temperature to above the *T*_g_ of the PLGA shell (approximately 46.2 °C) to enhance the polymer mobility and therefore drug diffusion. The in vivo experiments showed a remarkable synergistic bactericidal effect of the HM-Van-PPyNPs under the irradiation of an external NIR laser.

**Figure 8:**
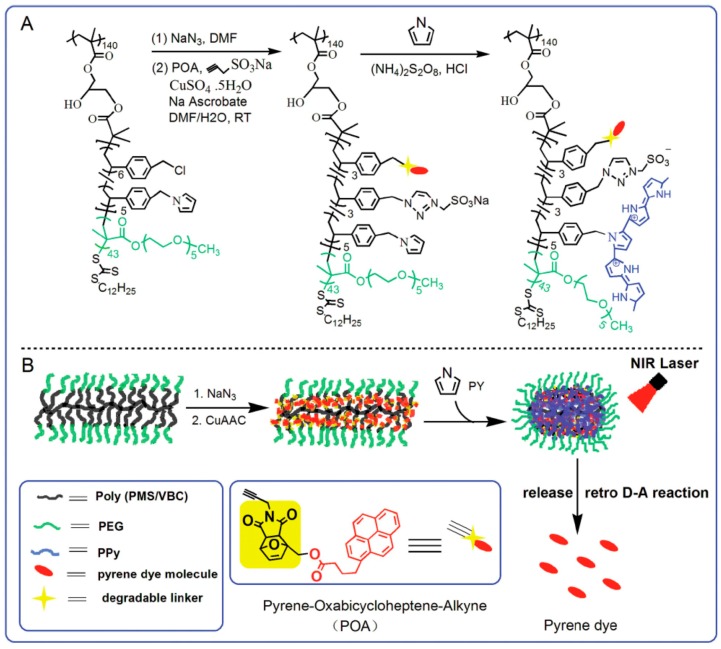
(**A**) Synthetic route of PEG-POA PPy NPs; (**B**) Graphic showing the synthesis of PEG-POA PPy NPs and photothermal-induced release of pyrene dye by the retro D-A reaction under NIR irradiation. Reproduced with permission from [101].

**Figure 9:**
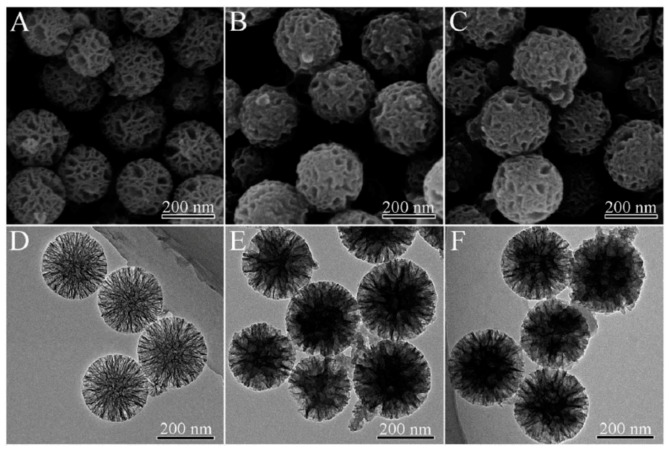
SEM (**A**–**C**) and TEM (**D**–**F**) images of DSNs–NH_2_ (**A**,**D**); PPy@DSNs–NH_2_ (**B**,**E**); and PPy@DSNs–PEG (**C**,**F**). Reproduced with permission from [102].

The loading of PPy NPs on the third-part matrix also can be achieved by in situ synthesis of PPy NPs in the third-part matrix. For example, Zhang et al. [101] synthesized a PEGlated pyrrole-containing bottlebrush copolymer by “graft-from” approach taking advantage of reversible addition-fragmentation chain transfer (RAFT) polymerization, as shown in Figure 8. A well-defined poly(glycidyl methacrylate) (PGM) was first synthesized by RAFT polymerization as the backbone. Next, 4-vinylbenzyl chloride (VBC) and 4-(pyrrolylmethyl)styrene (PMS) were randomly grafted onto the PGM backbone to form bottlebrush copolymers poly(GM-*g*-PMS/VBC) (with an average of 6VBC and 5PMS units). Then, a poly(ethylene glycol)methylether methacrylate (PEGMA) (with an average polymerization degree of 43) shell layer was introduced into the bottlebrush architecture to improve the water solubility and biocompatibility. The resultant PEGlated pyrrole-containing bottlebrush copolymer, poly(GM-*g*-PMS/VBC-PEGMA) was treated with NaN_3_ to introduce azido groups, and then sodium propynesulfonate and dye molecules (pyrene-oxabicycloheptenealkyne, POA), as the model drug, were coupled into the core section via a copper-catalyzed azide-alkyne cycloaddition (CuAAC) reaction. Finally, PPy NPs were in situ synthesized in the bottlebrush copolymer by the chemical oxidative polymerization of pyrrole monomer. The release behavior of pyrene under the irradiation of NIR laser indicated that PPy NPs acted not only as the efficient photothermal agent for photothermal therapy but also good controllers of a drug release system by retro Diels-Alder (retro D-A) reaction of the 6-exo-tetrahydrophthalhide unit in POA (Figure 9B, the yellow part of POA).

**Figure 10:**
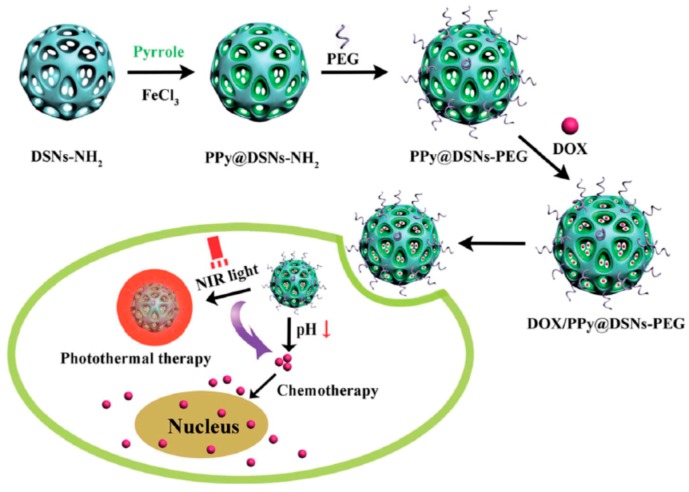
Schematic illustration of the preparation of DOX/PPy@DSNs–PEG for combined chemo-photothermal therapy. Reproduced with permission from [102].

Inorganic materials also can be used as the third-part matrix to combine the PPy NPs and other functional units. Chen et al. [102] synthesized a kind of mesoporous amino-functionalized dendrimer-like silica nanoparticles (DSNs–NH_2_) with an average diameter about 233 nm through the hydrolysis of tetraethyl orthosilicate (TEOS) and 3-aminopropyl-triethoxysilane (APTES) in an emulsion with a special composition [111], as shown in Figure 9A,D. The wrinkled sheets on the prepared DSNs–NH_2_ nanoparticle surface were grown in three dimensions forming the large pores. PPy chains were then in situ formed in the channels of DSNs–NH_2_ nanoparticles by the chemical oxidative polymerization of pyrrole to obtain the PPy@DSNs–NH_2_ nanoparticles, as shown in Figure 9B,E, with the decrease of the size of large pores and the thickening of the dendrimer-like skeleton. Finally, in order to improve the biocompatibility and stability in physiological conditions, PPy@DSNs–NH_2_ was reacted with poly(ethylene glycol) monomethyl ether with one end of carboxyl group (PEG–COOH) to obtain PEGylated PPy@DSNs–NH_2_ (PPy@DSNs–PEG), as shown in Figure 9C,F. The PPy@DSNs–PEG contained about 42.1 wt % of PPy, and had a BET surface area of 165.9 m^2^·g^−1^, an average pore size of 3.8 nm, and a pore volume of 0.34 cm^3^·g^−1^. The authors then loaded doxorubicin (DOX) as the model drug molecule in the mesopores of PPy@DSNs–PEG. The actual DOX loading efficiency can reach 16.08% due to the strong π–π stacking interactions between PPy and DOX. The release of DOX from DOX/PPy@DSNs–PEG can be controlled by pH and NIR light stimuli, as illustrated in Figure 10. Moreover, the PPy@DSNs–PEG has no visible cytotoxicity against U251 and U87 MG cells at the concentration of 7.8–500 μg·mL^−1^ and exhibits negligible hemolysis activity when the concentration ranged from 15.6 to 500 μg·mL^−1^. However, the photothermal conversion effect of PPy@DSNs–PEG under an 808 nm NIR irradiation can effectively kill those two kinds of cells. It should be noted that the combination of DOX/PPy@DSNs–PEG nanocomposite and the NIR irradiation could achieve better therapeutic efficacy than PTT or chemotherapy alone.

**Figure 11:**
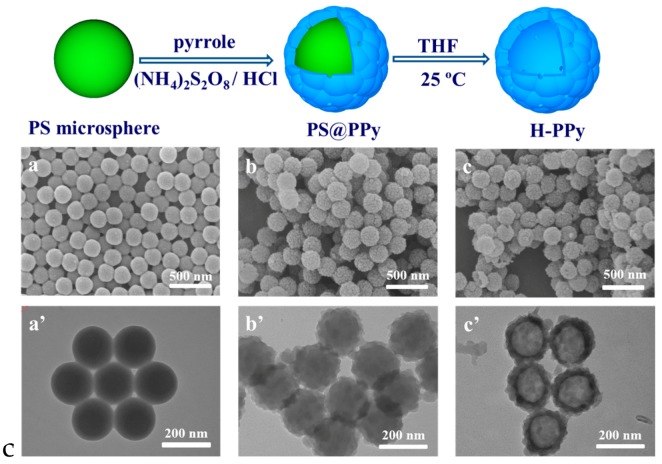
Schematic illustration of the synthesis of H-PPy microspheres. The SEM and TEM images of PS microspheres (**a**,**a’**), PS@PPy microspheres (**b**,**b’**), and H-PPy microspheres (**c**,**c’**). Reproduced with permission from [103].

**Figure 12:**
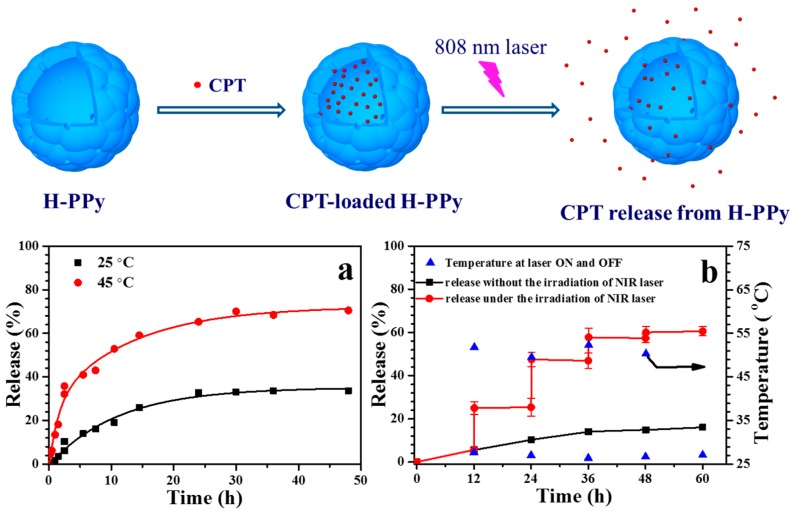
Schematic illustration of the loading and NIR-laser-triggered release of camptothecin (CPT) in H-PPy microspheres. (**a**) The releaseprofiles of CPT from H-PPy microspheres dispersed in the aqueous solution of DMSO (5% *v*/*v*) at 25 and 45 °C; (**b**) The release profile of CPT from H-PPy microspheres dispersed in the aqueous solution of DMSO (5% *v*/*v*) (0.1 mg·mL^−1^) under NIR laser (808 nm, 3.3 W·cm^−2^) irradiation for 5 min with an ON/OFF-mode every 12 h (-●-, left ordinate), and the corresponding temperature of the dispersion at every ON and OFF point (-▲-, right ordinate). As a control, the release profile of CPT in the same dispersion without NIR laser irradiation is also listed (-■-, left ordinate). Reproduced with permission from [103].

In addition to the above combination of PPy NPs and drug on a third-part matrix to achieve the synergistic dual chemo-photothermal therapy, researchers have made many attempts to design and synthesis on drug-loaded platforms using PPy as the matrix. Wang et al. [103] prepared raspberry-like hollow PPy (H-PPy) microspheres (~220 nm) using a sacrificial monodispersed polystyrene (PS) microsphere as the template, which was pre-prepared through the emulsion polymerization of styrene with PVP as the stabilizer. The negatively charged PS microspheres were first dispersed into the acidic solution of pyrrole. Since pyrrole molecules are positively charged in an acidic environment, they tend to be adsorbed on the surface of PS microspheres and polymerize into PPy NPs, resulting in the formation of raspberry-like PS@PPy microspheres, as shown in Figure 11. After the PS@PPy microspheres were immersed into THF to dissolve the PS cores, H-PPy microspheres were obtained. The H-PPy was found to have remarkable photothermal effect and good photostability and can load (S)-(+)-camptothecin (CPT) in the cavity through a diffusion and permeation process. The loading capacity of CPT can reach 0.14 mg/(mg H-PPy). The release of loaded CPT in H-PPy microspheres can be controlled by the irradiation of NIR laser (as shown in Figure 12). The in vitro experiment proved that the CPT-loaded H-PPy microspheres can exhibit excellent synergistic effect of chemotherapy and photothermal ablation on HeLa cells.

**Figure 13:**
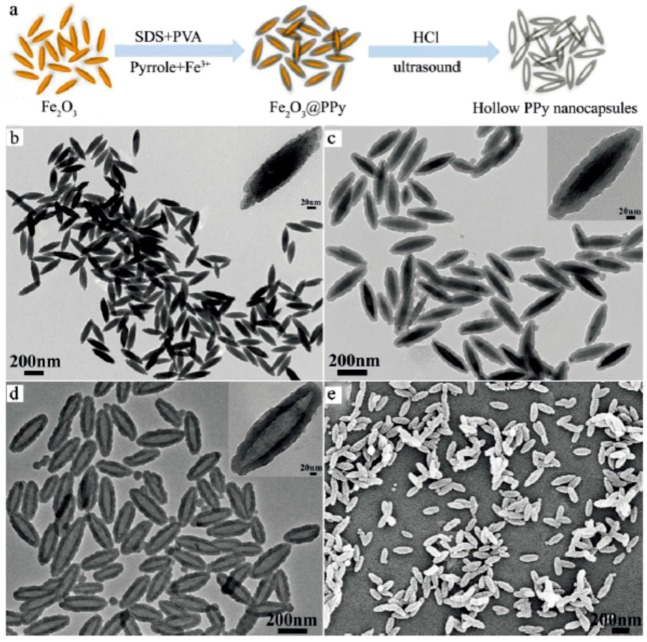
Schematic procedure for the synthesis of spindle-like PPy HNCs in (**a**) an aqueous solution; TEM images of (**b**) Fe_2_O_3_; (**c**) Fe_2_O_3_@PPy; and (**d**) PPy HNCs; SEM image of (**e**) PPy HNCs. Reproduced with permission from [104].

Similarly, hollow PPy drug carrier with different morphology can be fabricated when different templates are used. For example, spindle-like polypyrrole hollow nanocapsules (PPy HNCs) as multifunctional platforms for highly effective chemo–photothermal combination can be prepared using spindle-like Fe_2_O_3_ particles as the template, as shown in Figure 13 [104]. When DOX was loaded in PPy HNCs, the drug carrier platform can penetrate cells more rapidly and efficiently in comparison with the size-matched spherical PPy HNCs (155 nm, with 30 nm shell structure). The release of DOX from the spindle-like PPy HNCs is pH-sensitive and enhanced by the irradiation of NIR light. As a result, the DOX-loaded spindle-like PPy HNCs combined with the NIR light irradiation also exhibited a highly effective chemo-photothermal therapy on the Hep-G2 tumor model on BALB/c nude mice.

**Figure 14:**
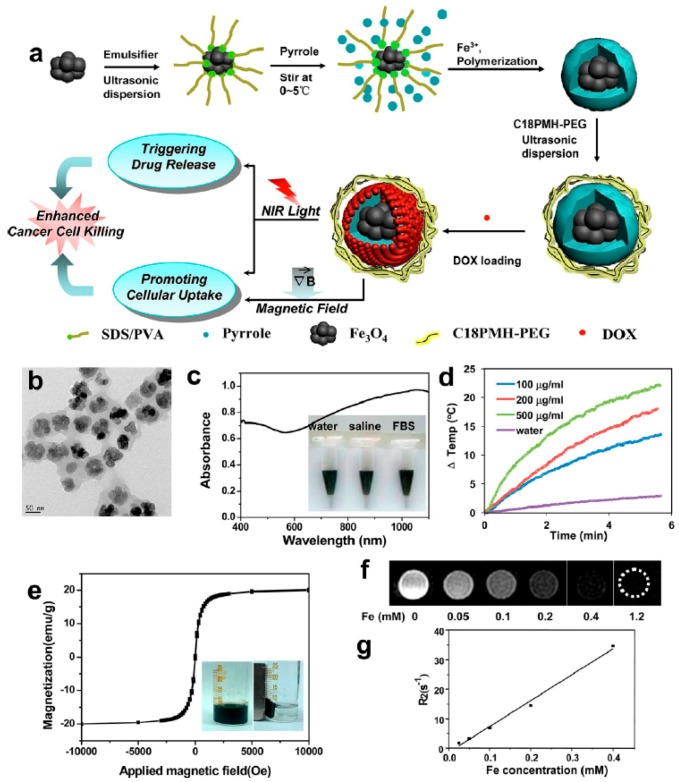
Preparation and characterization of Fe_3_O_4_@PPy–PEG nanocomposite. (**a**) Schematic illustration to show the synthesis of Fe_3_O_4_@PPy–PEG nanoparticles, the subsequent drug loading, and the remotely controlled cancer cell killing under dual physical stimuli. (**b**) TEM image of the synthesized Fe_3_O_4_@PPy–PEG nanoparticles; (**c**) UV–vis–NIR extinction spectra of Fe_3_O_4_@PPy–PEG nanoparticles in water (100 μg·mL^−1^). Inset: Photo of Fe_3_O_4_@PPy-PEG nanoparticles in different solutions including water, saline, and fetal bovine serum (FBS); (**d**) Temperature elevation of water and Fe_3_O_4_@PPy–PEG solution of different concentrations over a period of ~5.5 min under exposure of NIR light (808 nm, 0.75 W·cm^−2^) measured every 0.15 s using a digital thermocamera; (**e**) Field-dependentmagnetization loop of the Fe_3_O_4_@PPy–PEG sample. The absence of a hystersis loop suggested the superparamagnetic property of Fe_3_O_4_@PPy-PEG. Inset: Photos of Fe_3_O_4_@PPy–PEG solutions in the absence and presence of a magnet field; (**f**,**g**) T2-weighted MR images of the nanocomposite recorded using a 3 T MR scanner revealed a concentration-dependent darkening effect, showing a high transverse relaxivity (r2) of 87 mM^−1^·s^−1^. Reproduced with permission from [105].

Besides the hollow PPy NPs, PPy NPs having a heterogeneous solid core can also be used as promising multifunctional drug carrier. Wang et al. [105] prepared clusters of ultrasmall iron oxide magnetic nanoparticles and then dispersed them ultrasonically in the aqueous solution of pyrrole containing FeCl_3_, PVP, and the emulsifier dodecylbenzenesulfonic acid sodium salt (SDBS). After the in situ polymerization of pyrrole at room temperature, the iron oxide magnetic nanoparticles were coated with the NIR light-absorbing PPy polymer, forming core-shell Fe_3_O_4_@PPy NPs. In order to improve the stability of Fe_3_O_4_@PPy NPs in physiological solutions, amphiphilic PEG-grafted poly(maleic anhydride-*alt*-1-octadecene) (C18PMH-PEG) was coated on Fe_3_O_4_@PPy NPs through the physical adsorption, as shown in Figure 14. The obtained Fe_3_O_4_@PPy-PEG nanoparticles can effectively load DOX in the PPy shell. It was found that the intracellular uptake of Fe_3_O_4_@PPy-PEG-DOX was promoted by both external magnetic field and NIR laser irradiation. At the same time, the intracellular release of DOX from Fe_3_O_4_@PPy-PEG-DOX also could be triggered by the irradiation of NIR laser. The prominent in vivo synergistic therapeutic efficacy on 4T1 tumor cells was then revealed. Further, the tumor development after treatment can be well tracked in magnetic resonance imaging (MRI) directly using the magnetic Fe_3_O_4_ core as the T2 contrast agent. This work, no doubt, will encourage further explorations on the construction of novel multifunctional theranostic agents based on the NIR-absorbing nanomaterials for imaging-guided and remote control cancer therapy. It has been found that when the inorganic (metal or metal oxide) nanoparticles organize into certain regular structures, the performance of the nanoparticles can be effectively further improved [112,113,114]. For example, the assembly of nanoparticles into superparticles can enhance the photothermal performance significantly due to the optimization of electronic structures in the superparticles. To encapsulate more regular and stable Fe_3_O_4_ nanoparticles core, sodium dodecyl sulfate (SDS)-capped Fe_3_O_4_ superstructures were prepared by a microemulsion template technique before the coating of PPy [115].

Very recently, Attia et al. [106] reported a one-step synthesis of PEG-decorated hybrid iron oxide/PPy multifunctional nanoparticles loaded with hydrophobic drug (ketoprofen), which is based on the chemical oxidative polymerization of pyrrole in the presence of FeCl_3_, ketoprofen and PEGylated surfactants (Kolliphor^®^ HS 15, a mixture of free PEG 660 and PEG 660 hydroxystearate (MW 870 Da)). The reaction system involves a water phase containing FeCl_3_ and Kolliphor^®^ HS 15 and an oil phase, i.e., methanol containing pyrrole and ketoprofen. The organic phase was dropped into the aqueous phase under vigorous stirring. The polymerization reaction lasted 12 h at room temperature. Although the final product is an opaque aggregation of spherical nanoparticles with average particle size well below 50 nm, they still exhibit a controlled release behavior of the drug molecules, and the magnetic relaxometry studies confirmed their possible applications as potential contrast agent in the field of MRI.

An ideal theranostic agent for a safe and effective PTT treatment should function at various stages, i.e., to recognize the tumor site and the size before the treatment, to label the distribution of photothermal agents during the treatment, and to show the effectiveness after the treatment through appropriate imaging techniques. Therefore, the construction of novel theranostic agents integrated with the functions of both imaging, diagnosis, and therapy has attracted intensive research interests since it allows for therapeutic feedback through monitoring the status of injected agents and instantaneous responses to treatment [116,117].

**Figure 15:**
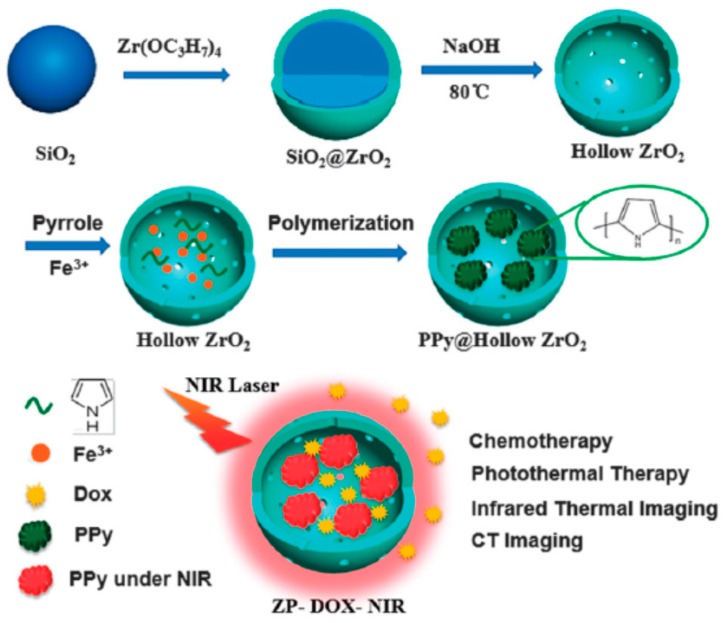
Synthetic scheme for ZrO_2_ hollow nanospheres via the template assisted method, in situ polymerization of PPy into the ZrO_2_ hollow nanospheres, and the principle of the integration of photothermal-chemo therapy, infrared thermal imaging, and CT imaging of ZrO_2_ hollow nanospheres loading with PPy and Dox under NIR laser irradiation. Reproduced with permission from [118].

**Figure 16:**
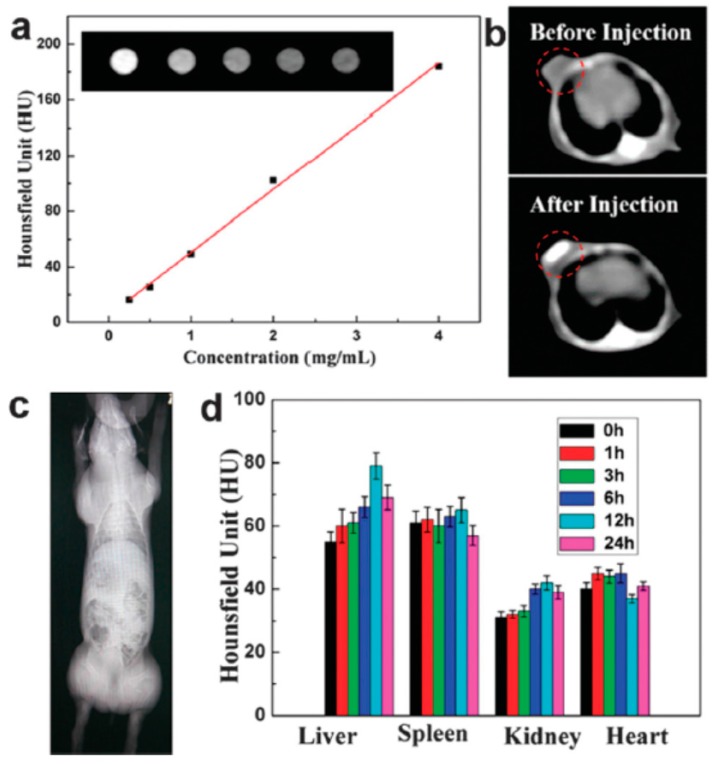
In vitro and in vivo evaluation of CT imaging efficiency of ZPs. (**a**) CT values and images (inset) of ZPs with different concentrations; (**b**) In vivo CT imaging of mice model before and post injection; (**c**) CT image of the whole mini swine model; (**d**) CT values of liver, spleen, kidney and heart of mini swine at different time points before and post injection. Reproduced with permission from [118].

Although zirconia is a biocompatible material, the structure adjustment and application of hollow zirconia as drug carrier have been seldom reported. Tan et al. [118] recently prepared uniform hollow ZrO_2_ nanospheres (210 ± 22 nm) with a shell thickness of 24 ± 4 nm as the nanocarriers. Due to the strong X-ray attenuation ability of Zr, the prepared hollow ZrO_2_ nanospheres have an inherent X-ray computed tomography (CT) imaging function. Then NIR light absorbing agent (PPy NPs) and anti-cancer drug (DOX) were both encapsulated into the hollow cavity. The synthesis idea is illustrated in Figure 15. Owing to their unique porous structures of PPy-loaded ZrO_2_ particles (ZP), the loading amount of DOX reached 30.7%. The NIR induced photothermal and chemotherapy of this nanoplatform were tested in vitro on HepG2 cells and in vivo on H22 tumors, respectively. The performance of the hollow ZP nanospheres as the contrast agent of CT imaging was also evaluated, as shown in Figure 16. Figure 16a shows the CT images and Hounsfield unit (HU) values in aqueous dispersions of ZPs with different concentrations, indicating a dramatic signal enhancement with the increase of ZP concentration. The HU value increased sharply from 39.14 ± 5.4 to 214.5 ± 10.5 HU after the injection of ZPs (20 mg·kg^−1^) in BALB/c mice bearing H22 tumors (Figure 16b), indicating a strong tumor contrast in the CT image. The real-time distribution of ZPs in vivo detected by CT imaging (Figure 16c,d) showed that the ZPs were mainly located in the spleen, liver, and kidney in vivo.

**Figure 17:**
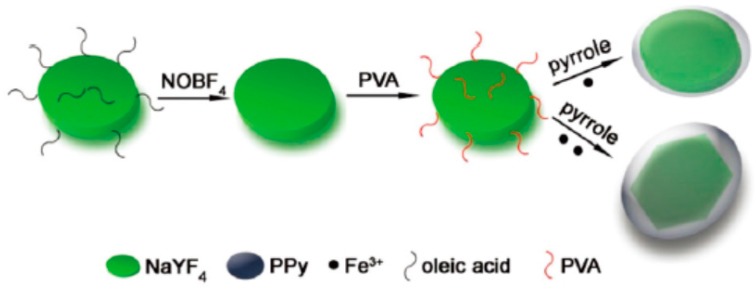
Schematic illustration of the synthetic route of NaYF_4_:Yb/Er@PPy core–shell nanoplates. Reproduced with permission from [119].

**Figure 18:**
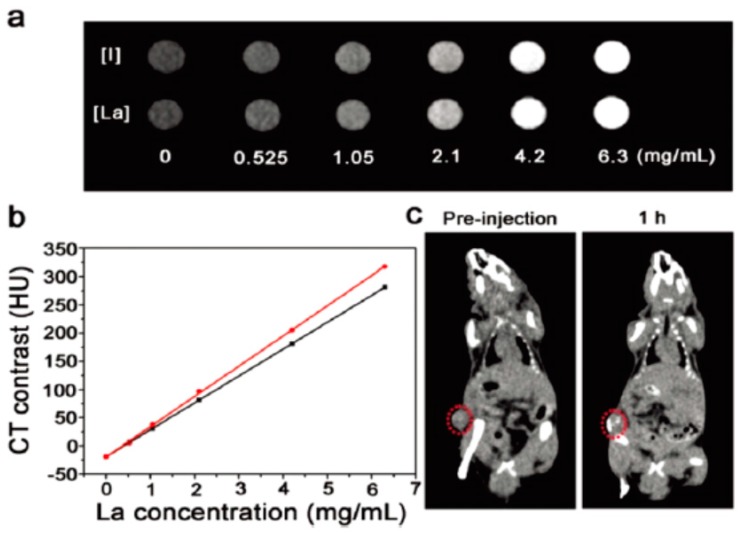
(**a**) In vitro CT images of iopromide and the dispersions of the NaYF_4_:Yb/Er@PPy nanoplates with different I or La concentrations; (**b**) CT value (HU) of iopromide (black line) and the NaYF_4_:Yb/Er@PPy nanoplates (red line) as a function of the concentration of I or La; (**c**) CT images of mice before and after intratumoral injection of the dispersion of the NaYF_4_:Yb/Er@PPy nanoplates. The position of tumors is marked by dotted circles. Reproduced with permission from [119].

Hang et al. [119] synthesized the oleic acid-modified NaYF_4_:Yb/Er nanoplates, and then converted them to PVA-functionalized NaYF_4_:Yb/Er nanoplates, finally made PPy successfully coat on the inorganic nano contrast agent, as shown in Figure 17. Generally, La-doped nanomaterials also possess better upconversion luminescence (UCL) imaging and MRI performance in addition to CT imaging. Compared with the fluorescence spectra of the dispersion of the NaYF_4_:Yb/Er and NaYF_4_:Yb/Er@PPy nanoplates, it can be seen that although the fluorescence was partly quenched after the thinner PPy shell was coated, the disc-shaped NaYF_4_:Yb/Er@PPy nanoplates still have enough strong upconversion fluorescence for UCL imaging. The in vitro CT imaging (Figure 18) shows the positive contrast enhancement of the CT signals with the increase of the nanoplate concentration. The slope of the linear equation for core–shell NaYF_4_:Yb/Er@PPy nanoplates is about 53.6 HU L·g^−1^, slightly higher than that of iopromide, suggesting that the core–shell NaYF_4_:Yb/Er@PPy nanoplates can be used as a CT contrast agent. Here, the core–shell NaYF_4_:Yb/Er@PPy nanoplates integrate triple imaging functions (infrared thermal imaging, UCL imaging, or CT imaging) and PTT for cancer cells. Similarly, FeWO_4_ nanoparticles could be explored as a potential dual-modal MRI/X-ray CT contrast agent. They can be combined with PPy using the same method to form a multifunctional therapy platform [120].

**Figure 19:**
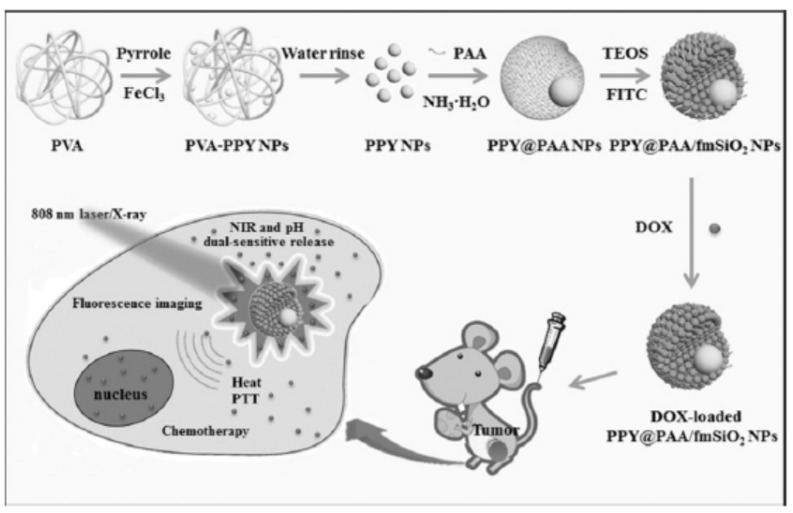
Schematic illustration for the synthesis of the multifunctional PPY@PAA/fmSiO_2_ NPs as NIR light and pH dual-stimuli responsive drug vehicles for fluorescence imaging and chemo-photothermal synergistic cancer therapy in vitro and in vivo. Reproduced with permission from [121].

Zhang et al. [121] also prepared core–shell multifunctional PPy@polyacrylic acid/fluorescent mesoporous silica nanoparticles (designated as PPY@PAA/fmSiO_2_ NPs) which integrate the high drug storage capacity, NIR light and pH dual-stimuli controlled drug release property, and strong fluorescence intensity, as shown in Figure 19. Uniform PPy NPs sized about 40 nm were firstly prepared in water through the chemical oxidation polymerization method using PVA as the stabilizer. Aqueous solution of PAA and ammonia was then mixed into the PPy aqueous solution, followed by the addition of isopropyl alcohol dropwise under vigorous stirring to obtain PPY@PAA NPs. Finally, TEOS and fluorescein isothiocyanate (FITC) conjugated with 3-aminopropytrimethoxysilane (APTMS) were introduced to form porous fmSiO_2_ shells on PPY@PAA NPs. The prepared PPY@PAA/fmSiO_2_ NPs are about 150 ± 5 nm in size, and emit bright green color excited by 365 nm UV light. When DOX was loaded into the PPY@PAA/fmSiO_2_ NPs, its release was stimulated by the change of pH and the irradiation of NIR light. Moreover, in vivo fluorescence imaging revealed the PPY@PAA/fmSiO_2_ NPs could be better uptaken by tumor cells because of the enhanced permeability and retention (EPR) effect after intravenous injection, which favors to visualize the location of tumors. An excellent tumor ablation effect, i.e., 95.6% of tumors were eliminated after the treatment of PPY@PAA/fmSiO_2_ NPs, was achieved, confirming that the chemo-photothermal therapy and imaging can be actually combined organically in one unit through a reasonable design.

**Figure 20:**
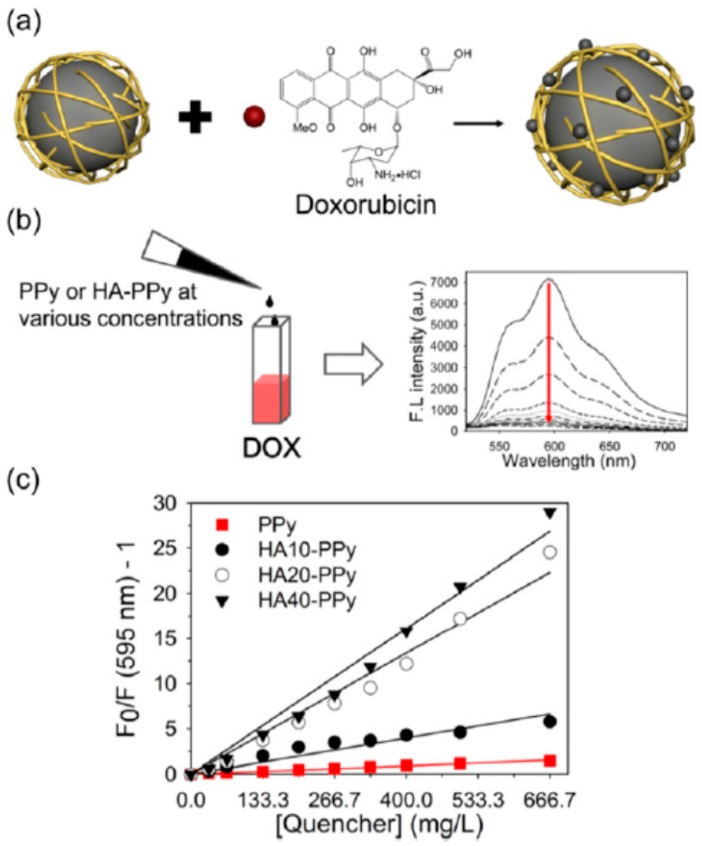
Fluorescence quenching of DOX by PPys and HA–PPys. (**a**) Illustration showing the formation of a complex between a nanoparticle and DOX, and the subsequent fluorescence quenching of DOX; (**b**) Fluorescence spectra of DOX solution (10 μg in 100 μL deionized water) mixed with HA–PPys at various concentrations (from top to bottom: 0, 10, 20, 40, 60, 80, 100, 120, 150, and 200 μg in 200 μL); (**c**) Stern–Volmer plots demonstrating the quenching of DOX fluorescence by HA–PPys. *K*sv of PPys (■), HA10-PPys (●), HA20-PPys (∘), and HA40-PPys (▼) was calculated to be 2.3, 9.9, 33.4, and 40.2 mL·mg^−1^, respectively. Reproduced with permission from [122].

Park et al. [122] prepared hyaluronic acid (HA, the molecular weight is 10, 20, and 40 kDa, respectively)-doped PPy NPs loaded with DOX (DOX@HA–PPys), as shown in Figure 20. It was found that replacing the stabilizer PVP on the surface of PPy with the more biocompatible HA favors not only to form uniform, spherical, and well-dispersed HA–PPys, but also to enhance the NIR photothermal effect of PPy. Moreover, the highly negatively charged surface of HA–PPys originated from the carboxylic acid groups could effectively adsorb the positively charged DOX via electrostatic interaction. It is interesting that a strong fluorescence quenching of DOX was observed (Figure 20b), which is believed to relate with the high molar extinction coefficients of PPy in the visible and NIR regions [55,87]. Calculated from the Stern–Volmer plots (Figure 20c), the quenching coefficients (*K*_sv_) for PPy, HA10-PPys, HA20-PPys, and HA40-PPys were 2.3, 9.9, 33.4, and 40.2 mL·mg^−1^, respectively. Investigations of the photothermal effect and the in vitro pH-dependent and light-induced release behavior of DOX@HA40-PPys demonstrated its potential as a photothermal and chemotherapeutic agent. Further, the chemo/photothermal dual therapy using DOX@HA–PPys was proved by the cytotoxicity assay on triple-negative breast cancer (TNBC) cells under various experimental conditions. In particular, the authors also illustrated the potential feasibility of the photothermal effect of HA40-PPys to overcome the chemoresistance in TNBC. Thus HA40-PPys can be considered as the nanocarrier combined with the functions of photothermal agent and fluorescence quencher.

**Figure 21:**
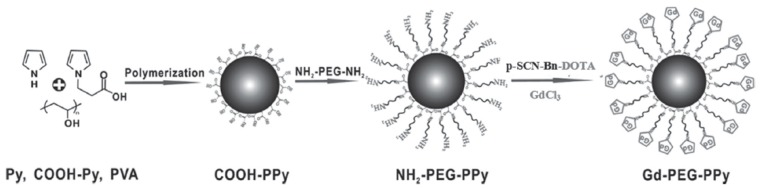
Schematic illustration of the fabrication process of Gd-PEG-PPy NPs. Reproduced with permission from [123].

Dual-modal MRI/photoacoustic imaging guided PTT also can be achieved based on PPy NPs. For example, Liang et al. [123] reported the fabrication of PEGylated PPy NPs conjugating gadolinium chelates (Gd-PEG-PPy NPs), as shown in Figure 21. First, the carboxylic-functionalized PPy NPs (COOH-PPy) were prepared from the mixture of pyrrole and pyrrole-1-propanoic acid (COOH-Py) at different molar ratios through a microemulsion polymerization method. Then, PEG-bis-amine was reacted with COOH-PPy to form PEG-coated and amino-functionalized PPy NPs (NH_2_-PEG-PPy). Finally, the NH_2_-PEG-PPy was covalently attached with gadolinium chelate (Gd-DOTA) (Gd-PEG-PPy). The Gd-PEG-PPy NPs were found to have a good biocompatibility to HUVECs, HeLa cells, T cells, and BMDC cells. But when combined with the NIR irradiation, the treatment of Gd-PEG-PPy NPs will cause the death of HeLa cells. Furthermore, Gd-PEG-PPy NPs also exhibited excellent in vivo MR and photoacoustic imaging capability. This work obviously provides another convincing demonstrational support on the feasibility of developing multifunctional nanomedicine platforms for the powerful cancer theranostics.

## 4. Conclusions and Future Perspective

In this review, the current synthesis routes of PPy NPs have been outlined. According to the basic polymerization principle of pyrrole, i.e., the cationic radical (oxidative) polymerization of pyrrole via a pseudo-polycondensation mechanism, PPy NPs are basically fabricated in an aqueous system dissolved with polymer stabilizers such as PVP and PVA. The most common oxidant is FeCl_3_. To obtain stable monodispersed PPy NPs, the synthesis conditions need to be carefully controlled. The molecular weight and concentration of the polymer stabilizer as well as the feed ratio of stabilizer to Fe^3+^ have great influence on the morphology of the final PPy NPs. On the other hand, new synthesis route free of chemical oxidant has been proposed, i.e., the water radiolysis under the exposure of high energy radiation, e.g., ^60^Co γ-ray, can directly produce the strong oxidative ·OH and H_2_O_2_ to initiate the polymerization of pyrrole. Nearly all the reported work indicate the PPy NPs also show remarkable NIR photothermal conversion performance and excellent biocompatibility, even coated with the polymer stabilizer.

But the photothermal therapy of PPy NPs is only the basic and primary application. Efforts on the design and fabrication of new PPy based multifunctional nanomaterials for cancer treatment in vivo have been intensively exerted. Loading drug and combination with various contrast agents are now the major subjects on the multifunctionalization of PPy nanoplatforms since the combination and visualization of therapies have become the inevitable trend of cancer treatment. This review summarized the very recent work on the preparation of PPy-based nanomaterials for imaging, and chemo-photothermal therapy. The current research results show an optimistic outlook of the emerging PPy-based nanomedicine platforms as an alternative future integrated cancer theranostics. However, more efforts and in-depth studies should be put forward urgently not only to develop more efficient and economic synthesis and construction methods for various integrated PPy-based theranostic nanoagents, but also to get the insight of the body circulation and the intracellular molecular mechanism of the photothermal effect of PPy nanomaterials, which is the key to open the gate to the practical remote monitoring chemo-photothermal synergistic cancer therapy.
